# Global Trends in Anaphylaxis Epidemiology and Clinical Implications

**DOI:** 10.1016/j.jaip.2019.11.027

**Published:** 2020-04

**Authors:** Paul J. Turner, Dianne E. Campbell, Megan S. Motosue, Ronna L. Campbell

**Affiliations:** a Section of Inflammation, Repair & Development, National Heart & Lung Institute, Imperial College London, London, United Kingdom; b Discipline of Child and Adolescent Health, University of Sydney, Sydney, NSW, Australia; c Department of Allergy and Immunology, Children's Hospital at Westmead, Sydney, NSW, Australia; d Department of Allergy and Immunology, University of Hawaii, John A. Burns School of Medicine, Honolulu, Hawaii; e Department of Emergency Medicine, Mayo Clinic College of Medicine and Science, Rochester, Minn

**Keywords:** Anaphylaxis, Biphasic, Epidemiology, Time trends, Food allergy, FAAN, Food Allergy and Anaphylaxis Network, NIAID, National Institute of Allergy and Infectious Diseases

## Abstract

The true global scale of anaphylaxis remains elusive, because many episodes occur in the community without presentation to health care facilities, and most regions have not yet developed reliable systems with which to monitor severe allergic events. The most robust data sets currently available are based largely on hospital admissions, which are limited by inherent issues of misdiagnosis, misclassification, and generalizability. Despite this, there is convincing evidence of a global increase in rates of all-cause anaphylaxis, driven largely by medication- and food-related anaphylaxis. There is no evidence of parallel increases in global all-cause anaphylaxis mortality, with surprisingly similar estimates for case-fatality rates at approximately 0.5% to 1% of fatal outcomes for hospitalizations due to anaphylaxis across several regions. Studying regional patterns of anaphylaxis to certain triggers have provided valuable insights into susceptibility and sensitizing events: for example, the link between the mAb cetuximab and allergy to mammalian meat. Likewise, data from published fatality registers can identify potentially modifiable risk factors that can be used to inform clinical practice, such as prevention of delayed epinephrine administration, correct posturing during anaphylaxis, special attention to populations at risk (such as the elderly on multiple medications), and use of venom immunotherapy in individuals at risk of insect-related anaphylaxis.

## Introduction

Anaphylaxis represents the more severe end of the spectrum of allergic reactions, and is most commonly triggered by medication, food, or insect stings. Measuring and evaluating epidemiological data related to episodes of anaphylaxis is an important means by which trends, burden of disease, and risk factors can be identified. Such information can highlight novel emerging allergens, changes in epidemiology, and risk-factor associations, which can in turn inform clinical practice and may prevent future severe reactions and fatalities.

Difficulties in the collection and interpretation of epidemiological anaphylaxis data must be acknowledged. These include variation in definitions of anaphylaxis across different regions of the world, logistical and coding issues related to collection of large health service data sets, and the inherent difficulties in collecting data for a disease state that largely occurs in the community, not within a hospital or health facility.

## Trends in Anaphylaxis Epidemiology

Hospital admissions data sets represent the largest and most robust data available to understand trends in anaphylaxis; however, they probably underestimate the true rate of anaphylaxis, because this frequently occurs in the community or outside of hospital settings, and only a minority of cases result in hospitalization.

**Figure 1:**
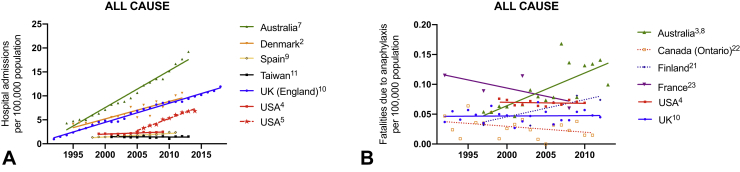
Time trends in hospital admissions (**A**) and fatalities (**B**) for all-cause anaphylaxis. Data from Motosue et al^2^ include all patients admitted to either an observation unit or a hospital ward. UK data relating to admissions after 2012 are previously unpublished but are obtained using identical methodology to that before 2012.^3^

**Figure 2:**
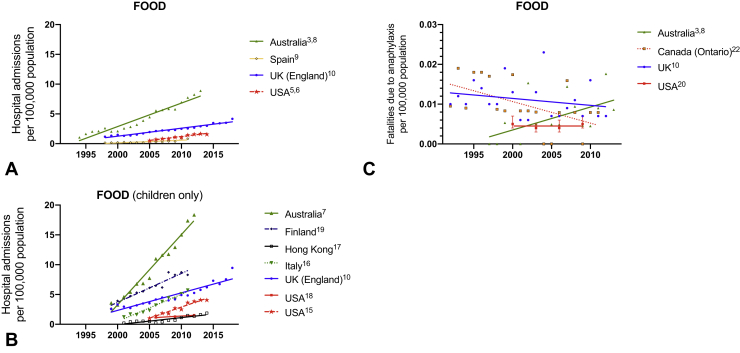
Time trends in hospital admissions (**A**, adults and children; **B**, children only) and fatalities (**C**) for food-induced anaphylaxis. Data from Motosue et al^4^ include all patients admitted to either an observation unit or a hospital ward. UK data relating to admissions after 2012 are previously unpublished but are obtained using identical methodology to that before 2012.^3^

**Figure 3:**
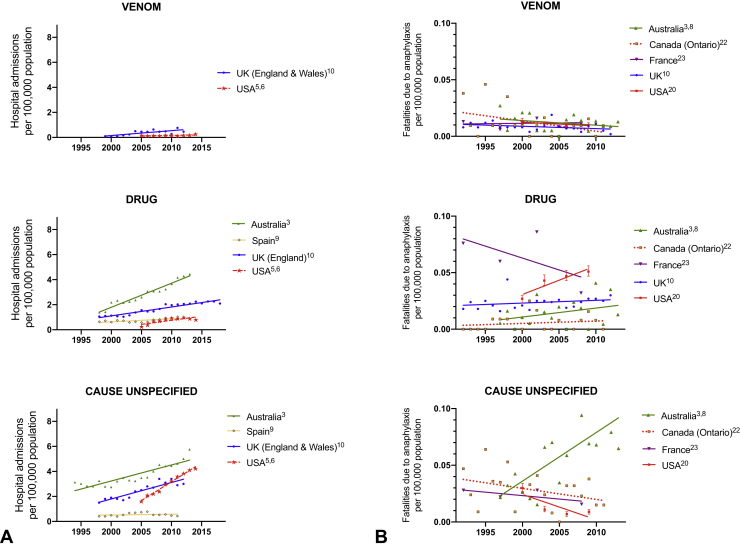
Time trends in hospital admissions (**A**) and fatalities (**B**) for anaphylaxis due to nonfood triggers, by agent (venom, medication, and “unspecified”). Data from Motosue et al^4^ include all patients admitted to either an observation unit or a hospital ward.

Anaphylaxis accounts for up to 0.26% of overall hospital admissions.^1^ In general, the literature reports global (United Kingdom, Europe, United States, Australia, New Zealand) increases in hospitalizations for anaphylaxis—both with respect to all-cause anaphylaxis (Figure 1) and by trigger (Figures 2 and 3)^2^, ^3^, ^4^, ^5^, ^6^, ^7^, ^8^, ^9^, ^10^; Taiwan appears to be an exception, where hospitalizations have not increased despite an increase in hospital referrals for anaphylaxis.^11^ Data are available relating to hospital *presentations* (rather than hospital *admissions*) from South Korea and New Zealand: all-cause anaphylaxis is estimated to have increased 1.7-fold over the period 2010 to 2014 in South Korea,^12^ most markedly in young children, whereas there has been a 2.8-fold increase in food-related anaphylaxis admissions in children in New Zealand between 2006 and 2015.^13^

There are significant differences in global anaphylaxis admission rates, with the highest rates in Australia and lower rates reported in Spain, Taiwan, and the United States. This may be due, in part, to different thresholds for observation in hospital after a reaction, and whether this occurs in an “observation unit,” which may or may not be coded as a hospital admission. Less than 20% of emergency presentations with anaphylaxis are admitted (either to an observation unit or to a hospital ward) in the United States,^4^ which could explain (at least in part) the lower rate of hospitalization in the United States (similarly, in Spain, most patients are discharged without hospitalization). This is in contrast to countries such as the United Kingdom where national guidelines recommend hospitalization for anaphylaxis, particularly in children at first presentation.^14^ In general, the increase in hospitalizations is predominantly due to food-related anaphylaxis, particularly in children,^3^^,^^6^, ^7^, ^8^, ^9^^,^^15^, ^16^, ^17^, ^18^, ^19^ although data are limited for nonfood allergens (Figure 2, *A* and *B*; Figure 3, *A*). Interestingly, rates of hospitalization are roughly equivalent in most regions (although highest in Australia), which implies that perhaps the threshold for in-patient observation is not particularly different between countries.

**Figure 4:**
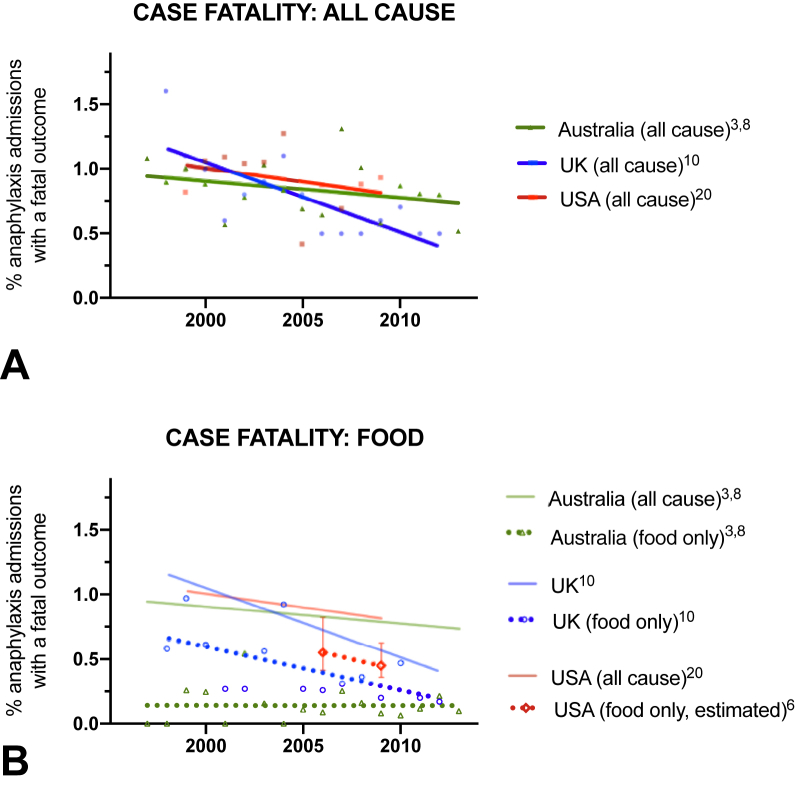
Incidence of fatal anaphylaxis expressed as a proportion of hospital admissions, for all cause (**A**) and due to food (**B**). Data for United States relating to case-fatality rate for food anaphylaxis estimated using data from Motosue et al^4^ and Jerschow et al.^20^

Despite this increase, there is little evidence that the overall rate of fatal outcomes has increased,^3^^,^^9^^,^^20^, ^21^, ^22^, ^23^ with the mortality rate declining in many regions. Furthermore, mortality seems similar in those regions where data are available, at around 0.5 to 1 fatality per million (population). The notable exception is Australia, where all-cause fatal anaphylaxis rates increased by 6.2% per annum from 1997 to 2013, predominantly due to food triggers.^9^ However, when these data are analyzed by case-fatality rate (proportion of cases admitted to hospital that result in a fatal outcome), mortality has fallen, including with respect to food-related fatal anaphylaxis in Australia (Figure 4).

## Trigger-Specific Epidemiology

Food represents the most common trigger for anaphylaxis admissions to hospital, but not the most common cause of anaphylaxis-related fatalities. Hospitalizations due to food-related anaphylaxis peak in the pediatric age range, but contribute significantly to adult admissions, where typically anaphylaxis admissions due to medication exceed those due to food by the sixth decade onwards. Mortality from food-related anaphylaxis is consistently lower than from other causes across all regions. This is in agreement with the observation that although food-induced anaphylaxis is relatively common, fatal outcomes are rare, with a reported incidence of 1.35 to 2.71 per million person-years.^24^ Curiously, the United States appears to have a lower mortality rate for food-related anaphylaxis (but not the proportion of hospital admissions resulting in fatal outcome, ie, case-fatality rate) compared with other regions, despite a higher mortality from all-cause anaphylaxis (in terms of both deaths per unit population and case-fatality rate) compared with Canada and the United Kingdom. The reasons for this are unclear, but may be due to miscoding: Ma et al^7^ reported that 75% anaphylaxis fatalities recorded between 1999 and 2009 in the United States were coded as “trigger unspecified.”

Both the United States and Australia have reported significant increases in fatality rates due to drug-induced anaphylaxis^9^^,^^20^ (Figure 3, *B*), which may represent an increasing tendency toward polypharmacy in an aging population—although there is no evidence that this has affected the case-fatality rate (Figure 4). Analysis of data from a national adverse drug reporting system in Vietnam over the period 2010 to 2016 found a significant increase in the rate of drug-related anaphylaxis, predominantly attributed to antibiotics.^25^ McCall et al recently analyzed time trends in US anaphylaxis-related hospitalizations in pregnant women between 2004 and 2014, to assess whether drug-related anaphylaxis had increased as a result of an increase in deliveries by cesarean section; reassuringly, the authors did not identify such an increase in this specific patient cohort.^26^

Insect-related anaphylaxis rates appear to have remained relatively stable (or decreasing) over many years, in comparison to medication and food. Although they represent a small proportion of hospital anaphylaxis admissions, they are relatively overrepresented in fatalities, underlying the seriousness of insect allergy. Potentially modifiable factors highlighted by anaphylaxis registries include delayed treatment due to rural location of incident, lack of preparedness for anaphylaxis, and lack of prior immunotherapy for venom allergy.^9^

## Novel and Emerging Allergens

Lipid transfer protein–associated food anaphylaxis is reported to be the most common cause of food anaphylaxis in adults in the Mediterranean region,^27^ with a north-to-south regional gradient in prevalence.^28^ It is also the most common trigger for exercise-associated, food-related anaphylaxis in this region.^29^ The epidemiology of this syndrome elsewhere is unclear: although sporadic case reports of lipid transfer protein–associated food anaphylaxis occur globally, it remains unclear exactly why this appears to be a largely Mediterranean phenomena, and whether rates are truly increasing.^30^

Allergy and anaphylaxis to the oligosaccharide galactosyl-α-(1,3)-galactose is an emerging cause of anaphylaxis in tick-endemic regions globally. It was the regional US epidemiology of anaphylaxis to cetuximab—concentrated in the southeastern US states of Tennessee, Arkansas, and South Carolina, with few anaphylaxis cases reported in Massachusetts and northern California—that led to the understanding that this form of anaphylaxis was related to prior sensitization to galactosyl-α-(1,3)-galactose via tick bites.^31^ In the form of nonprimate mammalian meat anaphylaxis, symptoms present with an “atypical” delay in onset from exposure to anaphylaxis, typically 3 to 6 hours after mammalian meat ingestion^32^^,^^33^ (although reactions up to 10 hours after exposure have been reported). Cases have been reported in most regions, including Australia,^34^ Japan,^35^ the United States,^30^ South America, Africa,^36^ and Europe, but it is unclear whether rates are increasing, with many historical cases likely to have been unrecognized and undiagnosed.^37^

mAbs are exponentially used in clinical practice to treat a wide range of diseases, and represent a novel therapeutic class that is increasingly associated with anaphylaxis, as recently reviewed.^38^ Ironically, the agent most commonly reported to trigger anaphylaxis is the anti-IgE mAb omalizumab^38^; however, systematic reporting and analysis of cofactors related to mAb-related anaphylaxis (aside from cetuximab) are currently lacking.

## Biphasic Anaphylaxis

Studies assessing the frequency of biphasic anaphylaxis have been undertaken worldwide.^39^ The true incidence of biphasic anaphylaxis remains unclear, hindered by the use of differing definitions of biphasic anaphylaxis. Studies evaluating the incidence of biphasic reactions have reported rates ranging from almost 20%^40^ to less than 1%.^41^ A meta-analysis of 27 studies, which included 4114 patients with anaphylaxis and 192 biphasic reactions, reported a biphasic reaction rate of 4.6% and a median time of onset of 11 (range, 0.2-72) hours.^39^ Risk factors associated with the development of a biphasic reaction have been difficult to identify. However, the data suggest that increased severity of the initial reaction,^42^^,^^43^ a wide pulse pressure^39^^,^^44^ at presentation, increased requirement for epinephrine to treat the initial reaction,^44^, ^45^, ^46^, ^47^ and delayed administration of epinephrine^44^^,^^45^^,^^48^ may increase the risk. Two systematic reviews^49^^,^^50^ failed to find evidence that corticosteroids reduce the risk of a biphasic reaction. Although no fatal reactions have been reported in contemporary studies evaluating biphasic anaphylaxis, approximately 20% to 55% of biphasic reactions are treated with epinephrine.^40^^,^^44^^,^^46^^,^^48^^,^^51^ In addition, intensive care unit admission may be required in 4% to 14% of patients.^51^^,^^52^

## Collecting and Interpreting Anaphylaxis Data—Pitfalls and Limitations

To effectively understand and learn from anaphylaxis data, it is important to understand the potential biases that can confound any inferences made. *Selection bias* occurs when anaphylaxis cases in any given data set differ systematically from general anaphylaxis, resulting in systematic differences that can impact interpretation. For example, there are a number of different data sets that can be used to monitor epidemiological trends, ranging from emergency department presentations to public data sets, health insurance databases, and anaphylaxis registries. These data sets may only capture cases presenting to specific health care facilities and not anaphylaxis in the community, which often does not present to health care professionals. Insurance databases may only include cases in insured individuals or those who present to specific facilities, and are therefore unlikely to represent all socioeconomic groups. Anaphylaxis registries are, by nature, retrospective and subject to reporting and recall bias, although one state in Australia now has mandatory anaphylaxis reporting of any case presenting to a hospital facility (but not to health care professionals outside hospital).^53^

Selection bias becomes a major confounder when evaluating severity: mild reactions may not be included because of nonpresentation to health care facilities, whereas severe (fatal) cases may occur prehospital and not be registered, or misclassified as being due to a different cause of death. There is little consensus as to what constitutes severe reactions: in a large prospective cohort of anaphylaxis presenting to an emergency department, 31% of cases had wheeze without any other major organ features.^42^ Such presentations might be coded as asthma rather than anaphylaxis. In contrast, nonanaphylaxis reactions that involve significant generalized urticaria and facial angioedema alone might be miscoded as anaphylaxis due to “visual” severity. A further concern, particularly with respect to drug-induced anaphylaxis, is underrecognition and underreporting potentially due to medicolegal concerns: many such cases result from patients being administered medication to which they were already known to be allergic.^9^^,^^54^

The other important bias to consider is *information bias*, which relates to misclassification of data. At a broad level, large data sets depend on medical coding, which are prone to misclassification.^24^^,^^55^ This issue is further confounded by differences in the definition of anaphylaxis,^14^ and the extent to which any definition is used to determine the coding, as recently highlighted by Wang et al.^56^ It is not uncommon, particularly in the emergency setting, for mild allergic reactions to be coded as anaphylaxis, and vice versa.^57^^,^^58^ For example, 48% of anaphylactic reactions in an emergency department in New York State were not coded as anaphylaxis despite fulfilling diagnostic criteria.^58^ It is also possible that nonallergic anaphylaxis mimics such as chronic idiopathic urticaria and hereditary angioedema could be misclassified as anaphylaxis. This can impact interpretation; for example, the inclusion of more mild cases as anaphylaxis will skew the result of any intervention toward a more favorable outcome (potential channeling bias).

Identifying specific anaphylaxis triggers is important; however, this information is frequently not collected with existing coding systems. Many coded anaphylaxis reactions are labeled “trigger unspecified,” which hampers the evaluation of risk factors for severe reactions and in assessing trends for specific triggers. For example, in an analysis of US data between 1999 and 2009, more than two-thirds of cases—hospitalizations and fatalities—were classified as “unspecified trigger.”^7^ The new *International Classification of Disease, Eleventh Revision* coding^59^ should improve this, although there may be initial difficulties in monitoring historical trends if different coding systems have to be integrated for analysis.

Caution is needed when interpreting mortality data: death certification is prone to miscoding (eg, cases of anaphylaxis may be miscoded as “severe asthma”).^60^ Most death certification follows World Allergy Organization guidelines, where one part gives the condition or sequence of conditions leading directly to death, a second section gives details of any associated conditions that contributed to the death, but are not part of the causal sequence. There have been examples of this resulting in gross overestimates in terms of fatalities due to allergy, if death certificates include allergy diagnoses even when they are not factors that contributed to the fatal outcome.

Monitoring the rate of hospital admissions is a frequent method used to assess epidemiology, but there are many factors that determine whether a particular patient is admitted to hospital or discharged. For example, guidance in the United Kingdom implemented in 2011 recommended that all children with food-related allergic reactions be admitted to the hospital after presentation to the emergency department, which might have caused an artifactual increase in rates of hospitalization.^3^ Using prescription data for epinephrine autoinjectors as a surrogate for prevalence is also subject to similar external “modifiers,” because changes in prescription patterns cannot solely be attributed to changes in prevalence.

Despite limitations, analyzing changes in anaphylaxis epidemiology over time is an important tool for clinicians, researchers, and those advocating for improvements in health policy to address the burden of disease. The effect of bias can be mitigated in part by the use of the same methodology to compare trends in any given data set—so although there may be issues relating to information bias, if these are constant over the time period under study in any given data set, then underlying trends are likely to be real even if the biases confound any comparison between different data sets.

## Lessons For Improvement in Diagnosis and Management

Recognition of anaphylaxis can be difficult: this is confounded by differences in diagnostic criteria. This is particularly true for food-induced anaphylaxis: according to the National Institute of Allergy and Infectious Diseases/Food Allergy and Anaphylaxis Network (NIAID/FAAN) criteria^61^ (subsequently adopted by the World Allergy Organization^62^), a food-induced reaction with hives and vomiting could be consistent with anaphylaxis, but such a reaction (ie, skin and gut symptoms) would not be considered as anaphylaxis in the United Kingdom^63^ and Australia,^64^ in the absence of respiratory or cardiovascular symptoms. Furthermore, isolated respiratory reactions in the absence of skin or gut symptoms are not classified as anaphylaxis according to NIAID/FAAN criteria, despite this being a common presentation for fatal food anaphylaxis.^65^^,^^66^ For example, in the largest phase 3 study of oral immunotherapy performed to date (the PALISADE study), at least one-third of 551 participants received epinephrine during entry food challenge,^67^ but only 28 had reactions that met the NIAID/FAAN criteria for anaphylaxis.^68^ Interestingly, 35 subjects were treated for wheezing—7 more than those diagnosed with anaphylaxis—and at least 14 without anaphylaxis received multiple doses of epinephrine.^68^ These differences not only impact patient care practices but also have implications for service evaluation and research by confounding comparisons of reported incidence rates of anaphylaxis and epinephrine use due to differences in definition. Increased collaboration to create an international consensus is needed, to avoid these incongruities. The World Allergy Organization Anaphylaxis Committee has recently proposed a refinement of the NIAID/FAAN criteria and its rationale for doing so^69^ (Table I), to help achieve this important goal. Similarly, the implementation of *International Classification of Disease, Eleventh Revision* classification will also improve consistency of coding and facilitate future evaluation of epidemiological trends.

**Table I:** Amended criteria for the diagnosis of anaphylaxis, proposed by the World Allergy Organization Anaphylaxis Committee, 2019^63^

Anaphylaxis is highly likely when any 1 of the following 2 criteria is fulfilled:
1. Acute onset of an illness (minutes to several hours) with involvement of the skin, mucosal tissue, or both (eg, generalized hives, pruritus or flushing, and swollen lips-tongue-uvula)
And at least 1 of the following:
a. Respiratory compromise (eg, dyspnea, wheeze-bronchospasm, stridor, reduced PEF, and hypoxemia)
b. Reduced BP or associated symptoms of end-organ dysfunction (eg, hypotonia [collapse], syncope, and incontinence)
c. Severe gastrointestinal symptoms (eg, severe crampy abdominal pain and repetitive vomiting), especially after exposure to nonfood allergens
2. Acute onset of hypotension∗ or bronchospasm or laryngeal involvement† after exposure to a known or highly probable allergen for that patient (minutes to several hours‡), even in the absence of typical skin involvement

BP, Blood pressure; PEF, peak expiratory flow.

∗ Hypotension defined as a decrease in systolic BP >30% from that person's baseline, OR i. Infants and children younger than 10 y: systolic BP <(70 mm Hg + [2 × age in years]) ii. Adults: systolic BP <90 mm Hg.

† Laryngeal symptoms include stridor, vocal changes, and odynophagia.

‡ Most allergic reactions occur within 1 to 2 h of exposure, and usually much quicker. Reactions may be delayed for some food allergens (eg, galactosyl-α-(1,3)-galactose) or in the context of immunotherapy, occurring up to 10 h after ingestion.

Although largely a clinical diagnosis, biomarkers and specifically serum tryptase may support the diagnosis of anaphylaxis and aid in differentiating anaphylaxis from its mimics such as idiopathic systemic capillary leak syndrome and severe asthma, although serum tryptase level is also elevated in fatal asthma.^70^ This can be important in the context of monitoring trends for more severe reactions. Yet despite its recommendation in current guidelines,^62^^,^^71^ the role of tryptase in “real-world” practice remains debated. From a practical standpoint, serum tryptase is not readily available to emergency providers because it often takes several days for results to become available. Moreover, although the positive predictive value of serum tryptase is high (93%), the negative predictive value is low (17%)^72^ and may not be helpful, particularly for food-induced anaphylaxis when tryptase level is frequently *not* elevated. Other biomarkers (such as platelet-activating factor, cysteinyl leukotrienes, chemokine ligand-2) have been proposed^73^ but can be difficult to measure even under laboratory conditions. Future studies may need to consider examining the sensitivity and specificity of a combination of biomarkers.

With regard to anaphylaxis management, although epinephrine remains the first-line treatment, glucocorticoids and antihistamines including both H1- and H2-antihistamines are often recommended as second-line treatment (although the latest European Academy of Allergy and Clinical Immunology guidelines relegate antihistamines to a third-line measure to help relieve cutaneous symptoms, due to concerns that their use might delay the appropriate further administration of epinephrine or fluids during patient stabilization).^74^ The benefit of antihistamines and glucocorticoids in both acute management and prevention of biphasic reactions has not been established, and there is increasing evidence that glucocorticoids may be harmful rather than simply being of no benefit.^75^ What role, if any, glucocorticoids and antihistamines should have in anaphylaxis management needs further clarification, potentially through comparison of outcomes between different units/regions. Reports from regional fatal anaphylaxis registries have suggested that modifiable risk factors for severe and fatal anaphylaxis appear to include polypharmacy in the elderly, delayed administration of epinephrine, maintaining an upright posture (with dependent lower body) during anaphylaxis, failure to recognize history of medication allergies, and failure to undertake venom immunotherapy in at-risk venom-allergic individuals.^76^

Comprehensive management of patients who have had anaphylaxis can be complex, so partnerships between allergy specialists, emergency medicine, and primary care providers are necessary. Exploring the use of new tools, including the use of electronic medical records in providing structured ordered sets, discharge instructions, and automatic allergy referral system, may provide additional solutions to improve the diagnosis and management of anaphylaxis.
